# 4-Chloro-2-methyl-*N*-(3-methyl­phen­yl)benzene­sulfonamide

**DOI:** 10.1107/S1600536809051083

**Published:** 2009-12-04

**Authors:** B. Thimme Gowda, Sabine Foro, P. G. Nirmala, Hartmut Fuess

**Affiliations:** a Department of Chemistry, Mangalore University, Mangalagangotri 574 199, Mangalore, India; b Institute of Materials Science, Darmstadt University of Technology, Petersenstrasse 23, D-64287 Darmstadt, Germany

## Abstract

The N—H bond in the title compound, C_14_H_14_ClNO_2_S, the dihedral angle between the two benzene rings is 75.5 (1)°. The crystal structure features inversion-related dimers linked by pairs of N—H⋯O hydrogen bonds.

## Related literature

For the preparation, see: Savitha & Gowda (2006 ▶). For our study of the effect of substituents on the crystal structures of *N*-(ar­yl)aryl­sulfonamides, see: Gowda *et al.* (2009**a* ▶,*b* ▶,c* ▶). For related structures, see: Gelbrich *et al.* (2007 ▶); Perlovich *et al.* (2006 ▶).
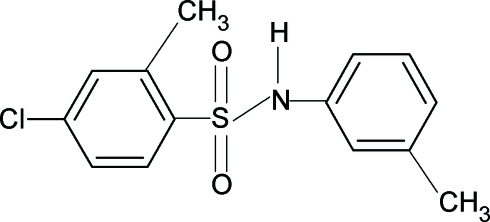


## Experimental

### 

#### Crystal data

C_14_H_14_ClNO_2_S
*M* *_r_* = 295.77
Monoclinic, 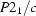

*a* = 7.8830 (7) Å
*b* = 11.602 (1) Å
*c* = 15.645 (2) Å
β = 90.593 (8)°
*V* = 1430.8 (3) Å^3^
*Z* = 4
Mo *K*α radiation
μ = 0.41 mm^−1^
*T* = 299 K
0.48 × 0.28 × 0.12 mm

#### Data collection

Oxford Diffraction Xcalibur diffractometer with a Sapphire CCD detector
Absorption correction: multi-scan (*CrysAlis RED*; Oxford Diffraction, 2009 ▶) *T*_min_ = 0.828, *T*_max_ = 0.953
5563 measured reflections
2925 independent reflections
1980 reflections with *I* > 2σ(*I*)
*R*_int_ = 0.014

#### Refinement

*R*[*F* ^2^ > 2σ(*F* ^2^)] = 0.052
*wR*(*F* ^2^) = 0.156
*S* = 1.03
2925 reflections
177 parameters
H atoms treated by a mixture of independent and constrained refinement
Δρ_max_ = 0.36 e Å^−3^
Δρ_min_ = −0.44 e Å^−3^

### 

Data collection: *CrysAlis CCD* (Oxford Diffraction, 2009 ▶); cell refinement: *CrysAlis RED* (Oxford Diffraction, 2009 ▶); data reduction: *CrysAlis RED*; program(s) used to solve structure: *SHELXS97* (Sheldrick, 2008 ▶); program(s) used to refine structure: *SHELXL97* (Sheldrick, 2008 ▶); molecular graphics: *PLATON* (Spek, 2009 ▶); software used to prepare material for publication: *SHELXL97*.

## Supplementary Material

Crystal structure: contains datablocks I, global. DOI: 10.1107/S1600536809051083/ci2978sup1.cif

Structure factors: contains datablocks I. DOI: 10.1107/S1600536809051083/ci2978Isup2.hkl

Additional supplementary materials:  crystallographic information; 3D view; checkCIF report

## Figures and Tables

**Table 1:** Hydrogen-bond geometry (Å, °)

*D*—H⋯*A*	*D*—H	H⋯*A*	*D*⋯*A*	*D*—H⋯*A*
N1—H1*N*⋯O2^i^	0.82 (4)	2.10 (4)	2.925 (3)	176 (3)

Symmetry code: (i) 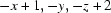.

**Fig. 1.**
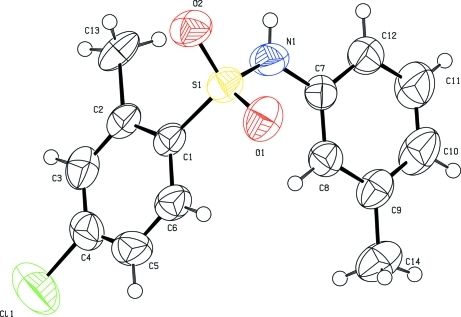
Molecular structure of the title compound, showing the atom labeling scheme. Displacement ellipsoids are drawn at the 50% probability level. H atoms are represented as small spheres of arbitrary radii.

**Fig. 2.**
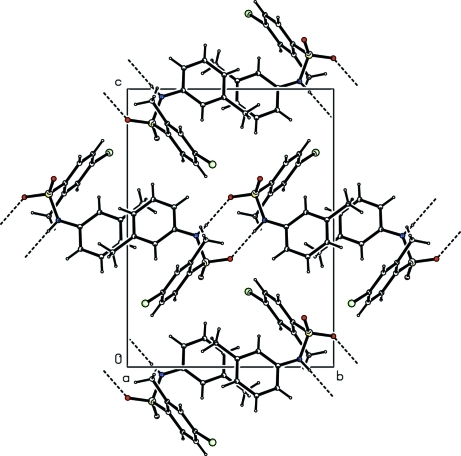
Molecular packing of the title compound with hydrogen bonding shown as dashed lines.

## supplementary crystallographic information

### Comment

As part of a study of the substituent effects on the structures of *N*-(aryl)arylsulfonamides (Gowda *et al.*, 2009*a,b,c*), in the present work the structure of 4-chloro-2-methyl-*N*- (3-methylphenyl)benzenesulfonamide (I) has been determined (Fig. 1). The conformation of the N—H bond is *anti* to the *meta*-methyl group in the aniline benzene ring. The molecule is bent at the *N* atom with the C1—S1—N1—C7 torsion angle of 77.2 (3)°, compared to the values of 73.0 (3)° in 4-chloro-2-methyl-*N*-(2-methylphenyl)benzenesulfonamide (II) (Gowda *et al.*, 2009*c*), 74.8°(4) in 2-methyl-4-chloro-*N*-(2-chlorophenyl)benzenesulfonamide (III) (Gowda *et al.*, 2009*b*) and -61.9 (4)° (molecule 1) and 69.7 (4)° (molecule 2) in the two independent molecules of 4-chloro-2-methyl-*N*- (phenyl)benzenesulfonamide (III) (Gowda *et al.*, 2009*a*). The two benzene rings are tilted relative to each other by 75.5 (1)°, compared to the values of 45.8 (1)° in (II), 45.5 (2)° in (III), and 86.6 (2)° (molecule 1) and 83.0 (2)° (molecule 2) in the two independent molecules of (IV). The other bond parameters in (I) are similar to those observed in (II), (III), (IV) and other aryl sulfonamides (Perlovich *et al.*, 2006; Gelbrich *et al.*, 2007). The crystal packing of molecules in (I) *via* N—H···O(S) hydrogen bonds (Table 1) is shown in Fig.2.

### Experimental

A solution of *m*-chlorotoluene (10 ml) in chloroform (40 ml) was treated dropwise with chlorosulfonic acid (25 ml) at 0 ° C. After the initial evolution of hydrogen chloride subsided, the reaction mixture was brought to room temperature and poured into crushed ice in a beaker. The chloroform layer was separated, washed with cold water and allowed to evaporate slowly. The residual 4-chloro-2-methylbenzenesulfonylchloride was treated with *m*-toluidine in the stoichiometric ratio and boiled for ten minutes. The reaction mixture was then cooled to room temperature and added to ice cold water (100 cc). The resultant solid 4-chloro-2-methyl-*N*- (3-methylphenyl)benzenesulfonamide was filtered under suction and washed thoroughly with cold water. It was then recrystallized to constant melting point from dilute ethanol. The purity of the compound was checked and characterized by recording its infrared and NMR spectra (Savitha & Gowda, 2006). The single crystals used in X-ray diffraction studies were grown in ethanolic solution by slow evaporation at room temperature.

### Refinement

The H atom of the NH group was located in a difference map and its positional parameters were refined with the N-H distance restrained to 0.82 (4) Å. The other H atoms were positioned with idealized geometry using a riding model [C–H = 0.93–0.96 Å]. All H atoms were refined with isotropic displacement parameters (set to 1.2 times of the *U*_eq_ of the parent atom).

### Crystal data

**Table:**

C_14_H_14_ClNO_2_S	*F*(000) = 616
*M**_r_* = 295.77	*D*_x_ = 1.373 Mg m^−^^3^
Monoclinic, *P*2_1_/*c*	Mo *K*α radiation, λ = 0.71073 Å
Hall symbol: -P 2ybc	Cell parameters from 2116 reflections
*a* = 7.8830 (7) Å	θ = 2.6–27.9°
*b* = 11.602 (1) Å	µ = 0.41 mm^−^^1^
*c* = 15.645 (2) Å	*T* = 299 K
β = 90.593 (8)°	Prism, colourless
*V* = 1430.8 (3) Å^3^	0.48 × 0.28 × 0.12 mm
*Z* = 4	

### Data collection

**Table:**

Oxford Diffraction Xcalibur diffractometer with a Sapphire CCD detector	2925 independent reflections
Radiation source: fine-focus sealed tube	1980 reflections with *I* > 2σ(*I*)
graphite	*R*_int_ = 0.014
Rotation method data acquisition using ω and φ scans	θ_max_ = 26.4°, θ_min_ = 2.6°
Absorption correction: multi-scan (*CrysAlis RED*; Oxford Diffraction, 2009)	*h* = −9→6
*T*_min_ = 0.828, *T*_max_ = 0.953	*k* = −11→14
5563 measured reflections	*l* = −19→18

### Refinement

**Table:**

Refinement on *F*^2^	Primary atom site location: structure-invariant direct methods
Least-squares matrix: full	Secondary atom site location: difference Fourier map
*R*[*F*^2^ > 2σ(*F*^2^)] = 0.052	Hydrogen site location: inferred from neighbouring sites
*wR*(*F*^2^) = 0.156	H atoms treated by a mixture of independent and constrained refinement
*S* = 1.03	*w* = 1/[σ^2^(*F*_o_^2^) + (0.0757*P*)^2^ + 0.6262*P*] where *P* = (*F*_o_^2^ + 2*F*_c_^2^)/3
2925 reflections	(Δ/σ)_max_ = 0.020
177 parameters	Δρ_max_ = 0.36 e Å^−^^3^
0 restraints	Δρ_min_ = −0.44 e Å^−^^3^

### Special details

**Table:**

Geometry. All e.s.d.'s (except the e.s.d. in the dihedral angle between two l.s. planes) are estimated using the full covariance matrix. The cell e.s.d.'s are taken into account individually in the estimation of e.s.d.'s in distances, angles and torsion angles; correlations between e.s.d.'s in cell parameters are only used when they are defined by crystal symmetry. An approximate (isotropic) treatment of cell e.s.d.'s is used for estimating e.s.d.'s involving l.s. planes.
Refinement. Refinement of *F*^2^ against ALL reflections. The weighted *R*-factor *wR* and goodness of fit *S* are based on *F*^2^, conventional *R*-factors *R* are based on *F*, with *F* set to zero for negative *F*^2^. The threshold expression of *F*^2^ > σ(*F*^2^) is used only for calculating *R*-factors(gt) *etc*. and is not relevant to the choice of reflections for refinement. *R*-factors based on *F*^2^ are statistically about twice as large as those based on *F*, and *R*- factors based on ALL data will be even larger.

### Fractional atomic coordinates and isotropic or equivalent isotropic displacement parameters (Å^2^)

**Table:**

	*x*	*y*	*z*	*U*_iso_*/*U*_eq_	
C1	0.6521 (3)	0.1989 (2)	0.83780 (16)	0.0484 (6)	
C2	0.8133 (4)	0.1933 (2)	0.87588 (18)	0.0552 (7)	
C3	0.9404 (4)	0.2588 (3)	0.8394 (2)	0.0679 (8)	
H3	1.0494	0.2563	0.8627	0.081*	
C4	0.9084 (4)	0.3278 (3)	0.7692 (2)	0.0675 (8)	
C5	0.7500 (5)	0.3331 (3)	0.7328 (2)	0.0725 (9)	
H5	0.7292	0.3802	0.6858	0.087*	
C6	0.6223 (4)	0.2679 (3)	0.76688 (18)	0.0616 (8)	
H6	0.5145	0.2700	0.7421	0.074*	
C7	0.3599 (3)	0.2701 (2)	0.99538 (18)	0.0509 (6)	
C8	0.3539 (3)	0.3658 (2)	0.94325 (19)	0.0584 (7)	
H8	0.4012	0.3622	0.8891	0.070*	
C9	0.2787 (4)	0.4672 (3)	0.9703 (2)	0.0630 (8)	
C10	0.2096 (5)	0.4692 (3)	1.0506 (2)	0.0882 (11)	
H10	0.1577	0.5362	1.0700	0.106*	
C11	0.2159 (5)	0.3740 (4)	1.1028 (2)	0.0929 (12)	
H11	0.1698	0.3776	1.1572	0.111*	
C12	0.2895 (4)	0.2737 (3)	1.07543 (19)	0.0663 (8)	
H12	0.2919	0.2090	1.1105	0.080*	
C13	0.8530 (4)	0.1198 (3)	0.9571 (2)	0.0703 (9)	
H13A	0.8313	0.0400	0.9451	0.084*	
H13B	0.7823	0.1448	1.0032	0.084*	
H13C	0.9701	0.1295	0.9732	0.084*	
C14	0.2762 (5)	0.5716 (3)	0.9131 (3)	0.0857 (11)	
H14A	0.2078	0.5560	0.8634	0.103*	
H14B	0.3898	0.5896	0.8961	0.103*	
H14C	0.2293	0.6359	0.9435	0.103*	
N1	0.4428 (4)	0.1662 (2)	0.97214 (17)	0.0692 (8)	
H1N	0.445 (4)	0.118 (3)	1.011 (2)	0.083*	
O1	0.3367 (3)	0.1432 (2)	0.82291 (15)	0.0752 (6)	
O2	0.5297 (3)	0.00166 (16)	0.88893 (14)	0.0708 (6)	
Cl1	1.07032 (16)	0.41278 (11)	0.72879 (9)	0.1237 (5)	
S1	0.47841 (9)	0.11958 (6)	0.87689 (5)	0.0572 (3)	

### Atomic displacement parameters (Å^2^)

**Table:**

	*U*^11^	*U*^22^	*U*^33^	*U*^12^	*U*^13^	*U*^23^
C1	0.0546 (15)	0.0446 (13)	0.0461 (14)	0.0116 (12)	−0.0020 (11)	−0.0040 (12)
C2	0.0599 (16)	0.0479 (15)	0.0576 (16)	0.0147 (13)	−0.0036 (13)	−0.0032 (13)
C3	0.0566 (17)	0.0603 (19)	0.087 (2)	0.0091 (15)	0.0030 (16)	−0.0136 (17)
C4	0.077 (2)	0.0543 (17)	0.071 (2)	0.0048 (15)	0.0271 (17)	−0.0090 (16)
C5	0.099 (3)	0.066 (2)	0.0528 (17)	0.0143 (19)	0.0131 (17)	0.0072 (15)
C6	0.0692 (19)	0.0651 (18)	0.0504 (16)	0.0146 (15)	−0.0034 (14)	0.0023 (14)
C7	0.0479 (14)	0.0486 (15)	0.0563 (16)	−0.0009 (11)	0.0032 (12)	−0.0036 (12)
C8	0.0590 (16)	0.0532 (17)	0.0631 (18)	0.0017 (13)	0.0068 (14)	−0.0016 (14)
C9	0.0591 (18)	0.0513 (16)	0.078 (2)	0.0032 (13)	−0.0098 (15)	−0.0089 (15)
C10	0.104 (3)	0.081 (3)	0.080 (3)	0.036 (2)	−0.004 (2)	−0.022 (2)
C11	0.107 (3)	0.108 (3)	0.064 (2)	0.036 (2)	0.015 (2)	−0.009 (2)
C12	0.0658 (18)	0.073 (2)	0.0604 (18)	0.0085 (15)	0.0035 (15)	0.0038 (15)
C13	0.0676 (19)	0.0651 (19)	0.078 (2)	0.0248 (15)	−0.0330 (16)	0.0020 (16)
C14	0.094 (3)	0.0524 (19)	0.111 (3)	0.0071 (18)	−0.014 (2)	0.0019 (19)
N1	0.095 (2)	0.0514 (15)	0.0613 (16)	0.0169 (14)	0.0169 (14)	0.0085 (12)
O1	0.0598 (12)	0.0789 (15)	0.0866 (16)	0.0024 (11)	−0.0155 (11)	−0.0093 (12)
O2	0.0932 (15)	0.0446 (11)	0.0749 (14)	0.0048 (10)	0.0039 (11)	−0.0041 (10)
Cl1	0.1293 (10)	0.1064 (9)	0.1367 (11)	−0.0264 (7)	0.0632 (8)	−0.0064 (7)
S1	0.0630 (5)	0.0485 (4)	0.0600 (5)	0.0044 (3)	−0.0008 (3)	−0.0033 (3)

### Geometric parameters (Å, °)

**Table:**

C1—C6	1.387 (4)	C9—C10	1.375 (5)
C1—C2	1.399 (4)	C9—C14	1.506 (5)
C1—S1	1.764 (3)	C10—C11	1.375 (5)
C2—C3	1.386 (4)	C10—H10	0.93
C2—C13	1.560 (4)	C11—C12	1.371 (5)
C3—C4	1.380 (5)	C11—H11	0.93
C3—H3	0.93	C12—H12	0.93
C4—C5	1.369 (5)	C13—H13A	0.96
C4—Cl1	1.737 (3)	C13—H13B	0.96
C5—C6	1.371 (4)	C13—H13C	0.96
C5—H5	0.93	C14—H14A	0.96
C6—H6	0.93	C14—H14B	0.96
C7—C12	1.375 (4)	C14—H14C	0.96
C7—C8	1.378 (4)	N1—S1	1.613 (3)
C7—N1	1.420 (3)	N1—H1N	0.82 (4)
C8—C9	1.386 (4)	O1—S1	1.420 (2)
C8—H8	0.93	O2—S1	1.438 (2)
			
C6—C1—C2	120.9 (3)	C9—C10—H10	119.4
C6—C1—S1	116.9 (2)	C12—C11—C10	120.6 (3)
C2—C1—S1	122.2 (2)	C12—C11—H11	119.7
C3—C2—C1	117.2 (3)	C10—C11—H11	119.7
C3—C2—C13	119.7 (3)	C11—C12—C7	119.1 (3)
C1—C2—C13	123.1 (3)	C11—C12—H12	120.4
C4—C3—C2	121.2 (3)	C7—C12—H12	120.4
C4—C3—H3	119.4	C2—C13—H13A	109.5
C2—C3—H3	119.4	C2—C13—H13B	109.5
C5—C4—C3	121.0 (3)	H13A—C13—H13B	109.5
C5—C4—Cl1	119.6 (3)	C2—C13—H13C	109.5
C3—C4—Cl1	119.3 (3)	H13A—C13—H13C	109.5
C4—C5—C6	119.0 (3)	H13B—C13—H13C	109.5
C4—C5—H5	120.5	C9—C14—H14A	109.5
C6—C5—H5	120.5	C9—C14—H14B	109.5
C5—C6—C1	120.7 (3)	H14A—C14—H14B	109.5
C5—C6—H6	119.7	C9—C14—H14C	109.5
C1—C6—H6	119.7	H14A—C14—H14C	109.5
C12—C7—C8	120.2 (3)	H14B—C14—H14C	109.5
C12—C7—N1	116.7 (3)	C7—N1—S1	127.3 (2)
C8—C7—N1	123.0 (3)	C7—N1—H1N	114 (3)
C7—C8—C9	121.0 (3)	S1—N1—H1N	116 (3)
C7—C8—H8	119.5	O1—S1—O2	118.67 (14)
C9—C8—H8	119.5	O1—S1—N1	109.96 (14)
C10—C9—C8	117.9 (3)	O2—S1—N1	104.43 (13)
C10—C9—C14	121.7 (3)	O1—S1—C1	107.55 (13)
C8—C9—C14	120.3 (3)	O2—S1—C1	108.88 (13)
C11—C10—C9	121.1 (3)	N1—S1—C1	106.78 (14)
C11—C10—H10	119.4		
			
C6—C1—C2—C3	−0.3 (4)	C8—C9—C10—C11	0.5 (5)
S1—C1—C2—C3	179.6 (2)	C14—C9—C10—C11	−178.6 (4)
C6—C1—C2—C13	178.3 (3)	C9—C10—C11—C12	−0.9 (6)
S1—C1—C2—C13	−1.7 (4)	C10—C11—C12—C7	1.1 (6)
C1—C2—C3—C4	0.8 (4)	C8—C7—C12—C11	−0.9 (5)
C13—C2—C3—C4	−177.8 (3)	N1—C7—C12—C11	176.6 (3)
C2—C3—C4—C5	−0.5 (5)	C12—C7—N1—S1	157.2 (2)
C2—C3—C4—Cl1	177.3 (2)	C8—C7—N1—S1	−25.5 (4)
C3—C4—C5—C6	−0.5 (5)	C7—N1—S1—O1	−39.2 (3)
Cl1—C4—C5—C6	−178.2 (2)	C7—N1—S1—O2	−167.5 (3)
C4—C5—C6—C1	1.0 (4)	C7—N1—S1—C1	77.2 (3)
C2—C1—C6—C5	−0.5 (4)	C6—C1—S1—O1	1.2 (2)
S1—C1—C6—C5	179.5 (2)	C2—C1—S1—O1	−178.8 (2)
C12—C7—C8—C9	0.5 (4)	C6—C1—S1—O2	130.9 (2)
N1—C7—C8—C9	−176.8 (3)	C2—C1—S1—O2	−49.0 (3)
C7—C8—C9—C10	−0.2 (4)	C6—C1—S1—N1	−116.8 (2)
C7—C8—C9—C14	178.9 (3)	C2—C1—S1—N1	63.2 (2)

### Hydrogen-bond geometry (Å, °)

**Table:**

*D*—H···*A*	*D*—H	H···*A*	*D*···*A*	*D*—H···*A*
N1—H1N···O2^i^	0.82 (4)	2.10 (4)	2.925 (3)	176 (3)

Symmetry codes: (i) −*x*+1, −*y*, −*z*+2.
